# Sealed with a Kick: A Case of Posttraumatic Coronary Artery Dissection and Cardiomyopathy

**DOI:** 10.1155/2012/208985

**Published:** 2012-12-12

**Authors:** Nithin Gottam, Sule Salami, Mahmoud Othman, James Torey, Howard Rosman, Andrzej Boguszewski

**Affiliations:** Department of Cardiology, St. John Hospital and Medical Center, 22101 Moross Road, Detroit, MI 48236, USA

## Abstract

Blunt chest trauma can lead to a variety of cardiac injuries, one of which is nonatherosclerotic myocardial infarction caused by intimal laceration and thrombotic process activation. Here we present a case of anterior myocardial infarction secondary to blunt trauma involving a kick to the chest.

## 1. Introduction

Blunt chest trauma can lead to a variety of different types of cardiac injury including: cardiac arrhythmias, myocardial muscle contusion, valvular disruption, coronary artery injury, cardiac chamber rupture, pericardial tamponade, and nonatherosclerotic myocardial infarction [1]. The reported incidence of injury varies from to 2 to 71% [2]. Non-atherosclerotic myocardial infarction is thought to occur through intimal laceration and thrombotic process activation secondary to the impact of the trauma and through acceleration and deceleration forces [3, 4]. Here we present a case of a young patient with acute myocardial infarction and cardiomyopathy after being kicked in the chest.

## 2. Case Report

A previously healthy 26-year-old Caucasian male presented to the emergency room (ER) with complaints of two-day duration of chest pain and shortness of breath after being kicked in the chest by his angry intoxicated girlfriend while he was lying in bed. The chest pain was initially pleuritic in nature, but by the next day changed in character to tightness in the chest, associated with shortness of breath. 

**Figure 1:**
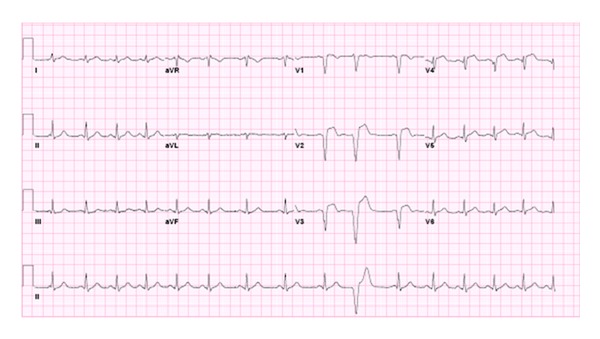
Sinus arrhythmia, with PVC. Anteroseptal infarct.

On initial examination, vital signs were 151/93 mm Hg, pulse of 95 beats/min, respiratory rate of 18 breaths/min, and temperature was 38.2°C. Pt was in moderate distress, noted to have trace area of erythema on anterior chest with no ecchymosis and mild palpable chest tenderness. Cardiac auscultation demonstrated no murmur, rub, or gallop. Pt was triaged and underwent CT angiography, which was negative for aortic dissection and pulmonary embolism. 12-lead electrocardiogram (EKG) demonstrated ST elevation in all precordial leads, most prominently in V2–V4. *Q* waves were present in V1–V3 (Figure 1). Given his continued chest pain, he was urgently taken to cardiac catheterization for coronary angiography. Initial cardiac Troponin-T level was 7.20 ng/L (normal <0.05). 

**Figure 2:**
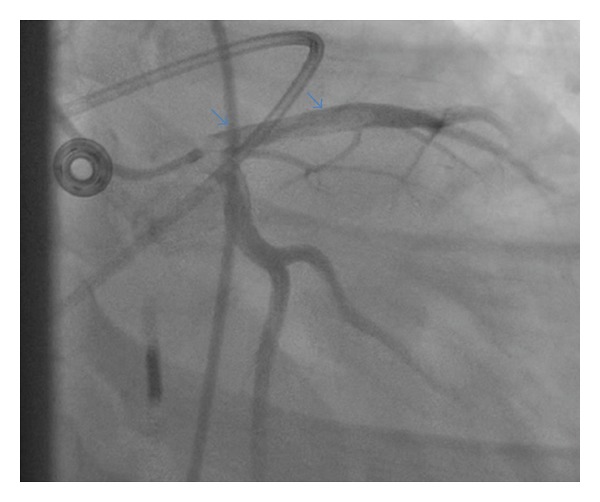
Coronary angiogram demonstrating a large filling defect in the proximal and mid portion of the left anterior descending (LAD) artery with distal TIMI 0 flow.

**Figure 3:**
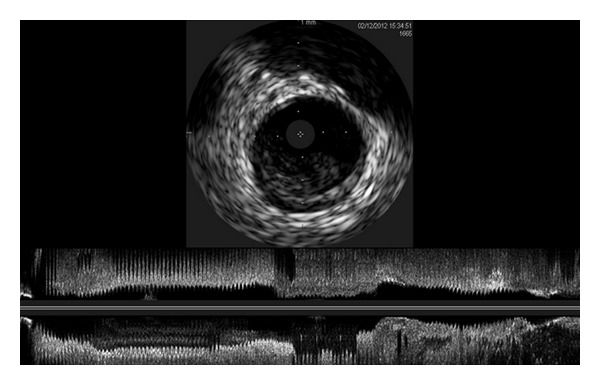
IVUS demonstrating significant residual thrombosis burden with LAD dissection.

The coronary angiogram demonstrated a filling defect in both the proximal and mid portions of the left anterior descending (LAD) artery with distal TIMI 0 flow (Figure 2). In addition, there was a small filling defect found in the proximal portion of the right coronary artery (RCA). He underwent several rounds of aspiration thrombectomy removing a large clot burden and afterwards underwent balloon angioplasty with a 2.5–20 mm balloon. Subsequently there was no myocardial uptake of contrast, which was treated with intracoronary nitroprusside and adenosine, after which intravascular ultrasound demonstrated significant residual thrombotic burden (Figure 3). In addition, intravascular ultrasound demonstrated a proximal soft hematoma, and distal intramural hematoma in the RCA. The left ventriculogram demonstrated a severely depressed left ventricular ejection fraction of 20% with associated apical akinesis. Pt underwent right heart catheterization, which demonstrated severely elevated wedge pressure. Due to the persistent no reflow state, an intra-aortic balloon pump (IABP) was inserted for hemodynamic support, and he was transferred to the cardiac intensive care unit (CICU) for observation. A bedside two-dimensional echocardiogram was performed prior to transfer to the CICU that demonstrated an ejection fraction of 20%, with preserved function in the basal segments only and akinesis everywhere else. No mass or thrombus was noted in the apex. 

Patient remained in CICU for 5 days with discontinuation of the IABP on day 4. He was subsequently discharged on day 9 of hospitalization. Repeat EKG and echocardiogram demonstrated continued *Q* waves with continued depressed left ventricular ejection fraction of 15%.

## 3. Discussion

Trauma-related myocardial infarction is caused by a variety of pathologic causes including: intimal tear, plaque rupture associated thrombosis, coronary artery disease, coronary artery spasm, coronary artery dissection, and compression due to epicardial hematoma [3]. The arteries most likely to be associated with trauma are the LAD (71.4%) and the RCA (19%) [4]. The LAD is more susceptible to injury due to its anterior location while the RCA has increased susceptibility secondary to acceleration and deceleration forces [4].

Diagnosis of myocardial infarction posttrauma is often delayed secondary to comorbid conditions associated with the trauma. In a review, by Christensen et al, it was noted that only 9% of MI was detected in the first 24 hours and 13% in the first week [4]. In addition to delayed presentation of MI other presentations include left ventricular dysfunction or coronary artery aneurysm [5].

Treatment approach to myocardial infarction after blunt chest trauma is determined by the associated injuries secondary to the trauma. Treatment options include conservative approach, use of thrombolytics, which are limited by bleeding limits [6], and by coronary angiography with percutaneous intervention. Coronary angiography may not demonstrate a coronary lesion, and the pathologic cause in these situations is thought to be coronary spasm and resolved thrombus [4]. In these situations, magnetic resonance imaging may be beneficial as it can reveal myocardial contusion and subendocardial/transmural myocardial infarction [7].

The presentation of our patient differed from other reported cases of blunt chest trauma leading to non-atherosclerotic myocardial infarction and dissection in that a single kick to the chest led to two coronary vessel dissections, anterior myocardial infarction, and presumed myocardial stunning. The power generated by a single kick is significantly less than the impact associated with moving vehicles. In addition, the kick's force was not magnified by acceleration and deceleration forces typically associated with trauma associated myocardial infarctions [4]. We present this case to demonstrate that varying degrees of trauma can cause non-atherosclerotic myocardial infarctions and myocardial stunning.
